# Outcomes reported in trials of methods for the induction of labour

**DOI:** 10.1186/1745-6215-16-S1-P4

**Published:** 2015-05-29

**Authors:** Nancy Medley, Zarko Alfirevic, Deborah M Caldwell, Sofia Dias, Therese Dowswell, Edna Keeney, Leanne Jones, Kate Navaratnam, Nicky Welton

**Affiliations:** 1 University of Liverpool, Department of Women’s and Children’s Health, Liverpool, L8 7SS, UK; 2 Bristol University, School of Social and Community Medicine, Bristol, BS82PS, UK

## Background

Labour inductions have increased steadily over the past two decades, with overall rates in many countries now exceeding 20% of all births. We have conducted a systematic review, network meta-analysis and cost-effectiveness analysis to determine which treatments for induction perform best on pre-specified safety and efficacy outcomes. This poster reports analysis of the outcomes reported in trials.

## Methods

We searched the Cochrane Pregnancy and Childbirth Group Database of Trials, populated by a generic search strategy identifying all pregnancy and postpartum trials recorded in CENTRAL, MEDLINE, EMBASE, NHS EED and CINAHL.

Randomised trials of all induction methods used in women at or near term (37 weeks) were included. Treatments included placebo, no treatment, prostaglandins, mechanical methods and alternative therapies. Data were extracted for seven pre-specified outcomes: vaginal delivery not achieved within 24 hours, uterine hyperstimulation with fetal heart rate (FHR) changes, caesarean section, serious neonatal morbidity or perinatal death, serious maternal morbidity or death, maternal satisfaction, and costs. Consumer representatives requested the additional outcome of instrumental delivery. The outcomes of interval to delivery, NICU admission and Apgar score were also added to inform clinical safety. Data on important effect modifiers (parity, previous CS, Bishop score, membrane status, multiple pregnancy, inpatient/outpatient) were also extracted.

## Results of the outcomes analysis

Apart from near universal reporting of caesarean section, three outcomes of hyperstimulation, Apgar < 7 at 5 minutes and instrumental delivery were reported in almost half of the review’s included studies. Just 5% of trials reported maternal satisfaction, and only 10% of trials reported costs. 23% of trials reported efficacy as measured in vaginal delivery within 24 hours. Equally surprising was the lack of safety data. 21% of trials reported neonatal death, and just 12% of trials reported serious maternal morbidity or death. 36% of trials reported NICU admission. Efficacy as measured in interval-to-delivery was reported in 55% of a subset of our included trials, but intervals were so variously defined that work is ongoing.

## Conclusions

There is little consensus on the most important outcomes for induction of labour trials. The next step to improve the reporting of efficacy and safety outcomes in induction of labour trials surely must be the agreement of a core outcome set. This process must include input from both clinicians and women. Once essential outcomes are agreed, standard measures must also be decided. Even for frequently reported outcomes the diversity of measures makes evidence synthesis extremely difficult.

